# Evaluating real-patient learning in medical education – Hungarian validation of the Manchester Clinical Placement Index

**DOI:** 10.3389/fmed.2023.1265804

**Published:** 2023-11-27

**Authors:** Szabolcs Fábry, Sándor Rózsa, Csenge Hargittay, Petra Kristóf, Ágnes Szélvári, Krisztián Vörös, Péter Torzsa, Endre Németh, Timothy Dornan, Ajándék Eőry

**Affiliations:** ^1^Heart and Vascular Center, Semmelweis University, Budapest, Hungary; ^2^Department of Anaesthesiology and Intensive Therapy, Semmelweis University, Budapest, Hungary; ^3^Department of Personality and Health Psychology, Károli Gáspár University of the Reformed Church, Budapest, Hungary; ^4^Department of Family Medicine, Semmelweis University, Budapest, Hungary; ^5^Faculty of Medicine, Semmelweis University, Budapest, Hungary; ^6^Centre for Medical Education, Queen’s University Belfast, Belfast, United Kingdom

**Keywords:** medical education, real patient learning, workplace learning, undergraduate, preclinical, bifactor analysis, Manchester Clinical Placement Index, validation

## Abstract

**Introduction:**

The Manchester Clinical Placement Index (MCPI) is an instrument to measure medical undergraduates’ real-patient learning in communities of practice both in hospital and in GP placements. Its suitability to evaluate the quality of placement learning environments has been validated in an English-language context; however, there is a lack of evidence for its applicability in other languages. Our aim was to thoroughly explore the factor structure and the key psychometric properties of the Hungarian language version.

**Methods:**

MCPI is an 8-item, mixed-method instrument which evaluates the quality of clinical placements as represented by the leadership, reception, supportiveness, facilities and organization of the placement (learning environment) as well as instruction, observation and feedback (training) on 7-point Likert scales with options for free-text comments on the strengths and weaknesses of the given placement on any of the items. We collected data online from medical students in their preclinical (1st, 2nd) as well as clinical years (4th, 5th) in a cross-sectional design in the academic years 2019–2020 and 2021–2022, by the end of their clinical placements. Our sample comprises data from 748 medical students. Exploratory and confirmatory factor analyses were performed, and higher-order factors were tested.

**Results:**

Although a bifactor model gave the best model fit (RMSEA = 0.024, CFI = 0.999, and TLI = 0.998), a high explained common variance (ECV = 0.82) and reliability coefficients (ωH = 0.87) for the general factor suggested that the Hungarian version of the MCPI could be considered unidimensional. Individual application of either of the subscales was not supported statistically due to their low reliabilities.

**Discussion:**

The Hungarian language version of MCPI proved to be a valid unidimensional instrument to measure the quality of undergraduate medical placements. The previously reported subscales were not robust enough, in the Hungarian context, to distinguish, statistically, the quality of learning environments from the training provided within those environments. This does not, however, preclude formative use of the subscales for quality improvement purposes.

## 1 Introduction

Undergraduate medical education is intended to equip students with general knowledge and skills needed for specialty training by supporting a “progressive and developmental, participatory, and situated and distributed” type of learning (1); however, contemporary medical education does not always prepare students adequately for the realities of practice (2). Burnout affects 15–41% of resident physicians according to a recent meta-analysis (3) and a lack of contextual knowledge (learning in the complex context of supportive clinical practice) contributes to ill-preparedness and early-career clinical errors (4). The stress that results from this is, arguably, a cause of burnout. Social features of workplaces (e.g., clinical leadership and a positive workplace climate), on the other hand, contribute significantly to a sense of thriving in junior doctors (5).

Theorists have tended to conceptualize experiential learning as an individual mental process, in which learners construct knowledge and personal meaning (6); however, social, and cultural interactions inevitably influence knowledge construction (6, 7). Medical students learn in communities of practice (COP) where informal learning from real patients exercises at least as strong an influence on students’ professional identity as formal education (6). Supervisors provide three types of support that foster the workplace learning that occurs in clerkships COPs. By providing affective support, they create learning environments with warm climates. Within these environments, they provide pedagogic support by instructing students how to apply skills to real patients, supervising their attempts and giving feedback on their performance. Organizational support, finally, creates preconditions for students to participate effectively in practice and learn from real patients (8). This nexus of conditions, processes, and complex learning outcomes is termed Experience based learning (7, 9).

To evaluate experience-based learning, Dornan and colleagues developed the Manchester Clinical Placement Index (MCPI) which measures the quality of learning environments, as judged by the support students receive from their preceptors during real patient learning in hospital and primary care COPs (10). MCPI contains numerical as well as free-text answer options and comprises 8 items which can be assessed as an aggregate measure (10). When validated in English language contexts, the originators of MCPI reported the existence of two independent subscales: first, five items that measure the affective support provided by learning environments as represented by the reception of students at the start of the placement, the supportiveness of people, and the quality of organization, leadership, and facilities; second, three items that evaluate the quality of training as represented by supervisors’ provision of instruction, observation and feedback (7, 11).

The validity of MCPI rests on ‘generic’ experiential learning theories (6, 7) as well as extensive research on medical undergraduates’ real patient learning (9, 12, 13). Its empirical validation showed equivalent discrimination between placements to the 50-item Dundee Ready Education Environment Measure (DREEM) (11, 14), which had been recommended as the instrument of choice to measure the quality of undergraduate medical learning environments (15). In addition to having as good or better psychometric properties as DREEM, MCPI’s mixed-method format gives it a unique advantage in comparison to purely numerical scales. The option for students to give free-text information to augment each numerical rating gives MCPI formative as well as summative properties. It allows continuous monitoring of the support given to students’ real patient learning in the whole span of curricula: from preclinical through clerkship years to graduation, makes it possible to quality-improve whole clinical curricula using data provided by students themselves, whose validity rests on both experiential learning theory and empirical research.

Until now, the formative benefits of MCPI have been restricted to the English-language cultures in which it was validated and has mainly been used. Given the persuasive arguments for its wider use, it is timely to explore the transferability of this tool to cultures where medical education is delivered in other languages. To explore the possibility of measuring the quality of the learning environment with an approved measurement tool is a prerequisite of the internationalization of medical education supporting the development of common standards, investigating cultural similarities and differences and their effects on education, and improving the quality and assessment of education at an international level. Hungary adopted MCPI early and has extensive experience of using it in a language other than English. Our aim, therefore, was to explore the psychometric properties (factor structure and reliability) of MCPI in a leading Hungarian medical school, as used by students in both preclinical and clerkship years, and in both GP and hospital placements. We capitalized on the qualitative as well as quantitative design of MCPI by choosing a mixed method study design.

## 2 Materials and methods

### 2.1 Study design and procedure

A single center, cross sectional study was conducted in the academic years 2019–2020 and 2021–2022 at Semmelweis University, Budapest.

#### 2.1.1 Procedure

In the year 2019–2020, second-year medical students – participants in the mandatory “Introduction to Clinical Medicine” placement – filled in an online version of MCPI in Hungarian, containing only a unique individual student passcode and the name of their preceptor. In year 2021–2022 data collection was extended to fourth- (general practice) and fifth year (anesthesiology and intensive therapy) medical students and the metadata were extended to include self-reported gender, age, and academic year. Questionnaires were circulated online using the university official mailing system at the end of the course (preclinical and fifth year students) or on paper during the closing lecture after the fourth-year primary care practical. The invitation letter accompanying the questionnaire stated that the Family Medicine Department in which students had just finished their placement aimed to collect feedback on how their preceptors and the community of practice supported their learning on real patients, with the double aim of improving the quality of workplace learning and conducting research into how to do so most effectively in future. All enrolled students were approached to fill the questionnaire. Quantitative items were mandatory to complete but free-text answers were optional. Preclinical students were motivated to participate by being entered into a lottery for three stethoscopes, while students in the clinical years received no incentives. Participation was anonymous and voluntary and had been approved by the regional ethical committee (No. 243/2019).

### 2.2 Participants

A total of 748 students completed the survey and they were all included into the analyses. The majority of respondents were female (*n* = 467, 62.4%) and their mean age was 21.4 years (SD = 2.4). The youngest was 18 and the oldest 46. Participants’ curriculum years were: first year 41.2%; second year 25.4%; fourth year 24.9%; fifth year 8.6%.

### 2.3 Real patient learning characteristics in different settings of the study samples

The Introduction to Clinical Medicine placement is a small group, one-semester, weekly practical for students in their preclinical years, which takes place in GP practices at times when doctors are not consulting. One patient is asked for permission for a group of 7–8 medical students to take their history in the presence of their general practitioner (GP) preceptor. A written history-taking guide^1^ translated from English to Hungarian is provided in advance and the preceptors support students’ individual participation in the practical by, for example, helping them frame questions, discussing problems, and answering questions that arise from contact with the patient. The aim is for students to be clinically immersed and acquire skills that enable them to approach patients and ask permission to take their medical history during the clerkship years. In the fourth year, students participate in a five-day Family Practice course, including two full-time GP placement days during which they observe the clinical work of one GP preceptor in a one-to-one relationship. Fifth year students have three-week Intensive Therapy and Anesthesiology placements, where they are instructed to complete eight bed-side activities in Intensive Care Units, where they assess patients according to the Airway, Breathing, Circulation, Disability, Exposure approach.

### 2.4 Outcome measure

The Manchester Clinical Placement Index (MCPI) is a self-report instrument developed by Dornan et al. (10) to measure the quality of support to students’ real patient learning in communities of practices. Its 8 items can be used together to measure educational environment and can be used separately, 5 items measuring learning environment (leadership, reception, supportiveness of people, organization, and facilities of the placement) and the remaining three assessing the quality of training (instruction, observation, and feedback). The 8 items are rated using 7-point Likert Scales whose extremes are 0 and 6, where 0 means strongly disagree, 3 means neither agree nor disagree and 6 means strongly agree to the item. Additionally, students can opt to give free-text comments on the strengths and weaknesses of the placement related to each of the same 8 items. Numeric data can be summed up to give an overall point score. In addition, the learning environment subscale can be calculated by adding up the scores for leadership, reception, people (support), facilities and organization, multiplied by 100 and divided by 30%. The training subscale is calculated by adding up the point scores for instruction, observation and feedback, multiplied by 100 and divided by 18%. We formulated an additional item about the clearness of the instrument using yes/no answer and free-text option as well: “Did you experience any difficulty in interpreting any of the questionnaire items while filling up the instrument?”. Answering the quantitative MCPI items was mandatory while the free form parts were optional.

### 2.5 Qualitative analysis

Having observed collinearity between participants’ numerical responses to the observation and feedback items, we chose to augment the statistical analysis with a qualitative analysis, whose aim was to explore how participants’ responses to the wording of the instrument contributed to this collinearity. These responses ranged from single-word answers to paragraphs. Following standard qualitative analytical procedures, Sz.F.; P.K.; Cs.H.; A.Sz.; A.E. read all free text responses systematically, identified blocks of text that pertained to observation, feedback and the relationship between the two, and assigned provisional codes. They compared their coding schemas, further discussed them with E.N.; P. T.; and T. D. and agreed on a common one. They then examined the codes, identified themes that organized the codes into higher-level concepts that explained how participants’ comments constructed the relationship between observation and feedback, constantly comparing their interpretation against the original data, and agreeing on a final interpretation.

### 2.6 Statistical analysis

Descriptive statistics (mean, standard deviation, skewness, and kurtosis), internal consistency and multivariate normality (Mardia’s coefficients) were calculated. Internal consistency was measured using Cronbach’s alpha with a minimal reliability coefficient criterion of 0.7 (16).

To determine the scale’s internal structure, we took a two-step approach. First, we explored the factor structure and factor loadings of each item using exploratory factor analysis (EFA), and then we subjected the complete dataset to confirmatory factor analysis (CFA), having first performed the Kaiser-Meyer-Olkin (KMO) Measure of Sampling Adequacy to examine the strength of correlation between the variables, taking 0.8 as our criterion of acceptability. We used Bartlett’s test of Sphericity to test if the correlation matrix was an identity matrix and accepted if that was not an identity matrix (*p* < 0.05) (17). Since our data were not normally distributed, we performed a polychoric correlation matrix for ordinal data and weighted least squares mean–variance adjusted (WLSMV) parameter estimator for factor analysis.

The number of dimensions to be extracted was defined by conducting a parallel analysis, which compared the progressive eigenvalues from the given data matrix to those of a simulated data matrix using random data of the same size (18). As has been recommended (19), 500 random datasets were generated, and the 95th percentiles of the eigenvalues from these random datasets were compared to those of the actual dataset. If the eigenvalue of the actual data was greater than the corresponding eigenvalue of the random data, the number of factors was retained (19, 20). The latent structure of multivariate data was also identified and visualized by the Gaussian graphical model (21) and a community detection algorithm for weighted graphs (22). Exploratory Graph Analysis (EGA) was used to model the inverse of the variance–covariance matrix via the graphical lasso (glasso) regularization method (23).

Confirmatory factor analysis (CFA) was performed by using a robust estimator (the maximum likelihood estimation with robust standard errors and a mean- and variance- adjusted, MLMV) that appropriately corrects for the standard errors of the parameters. We evaluated model fit by calculating chi-square, degree of freedom, root mean square error of approximation (RMSEA<0.06), comparative fit index (CFI > 0.95), and Tucker-Lewis Index (TLI > 0.95) (17, 24).

Explained common variance (ECV), which is an index of multidimensionality attributable to the general factor and each of the two group factors, is the proportion of all common variance explained by that factor. For the general factor, this is simply “ECV.” For specific factors, the ECV_S computes the strength of a specific factor relative to all explained variance of all items, even those not loading on the specific factor of interest (25).

Coefficient Omega (ω) is a model-based estimate of internal reliability of the multidimensional composite. It measures the proportion of total score variance which can be attributed to all common factors. For the general factor, all items were considered; for specific factors, Coefficient Omega Subscale (ωS) measured the proportion of subscale score variance, that was uniquely due to that factor after controlling for the general factor. Coefficient Omega Hierarchical (ωH) “reflects the percentage of systematic variance in unit-weighted (raw) total scores that can be attributed to the individual differences on the general factor, when ωH is high (>0.80), total scores can be considered essentially unidimensional.” The subscale omega hierarchical, omegaHS (ωHS), “is an index reflecting the proportion of reliable systematic variance of a subscale score after partitioning out variability attributed to the general factor” (26).

According to Stucky et al. (27), individual explained common variance (IECV) measures the extent to which an item’s responses are accounted for by variation on the latent general dimension alone, and thus acts as an assessment of unidimensionality at the individual item level. Selecting items with large loadings on the general factor and IECV greater than 0.8 or 0.85 will typically yield a fairly unidimensional item set that reflects the content of the general dimension. SPSS 17.0, FACTOR (28), Shiny app^2^ and Mplus Version 8 (29) were used for all data analysis.

## 3 Results

### 3.1 Descriptive statistics

The mean item scores ranged from 4.57 ± 3.31 (item 6 – feedback) to 5.46 ± 0.94 (item 1 – leadership), and all the items showed positive asymmetry and platykurtic distribution (Table 1). The univariate skewness and kurtosis values of several items fell outside the acceptable limit (±2.00), indicating that the assumption of normality was violated. The (30) estimate of multivariate kurtosis indicated deviation of the item scores from multivariate normality (*p* < 0.05). Therefore, the polychoric correlation and weighted least square mean and variance adjusted (WLSMV) parameter estimator was considered suitable for performing factor analysis. The correlation matrix of all items showed that most of the items correlated highly. The correlations among items 5 (observation) and 6 (feedback) were nearly 0.80, which indicated that multicollinearity could be a problem (17). The reliability tests on the measures for Learning environment (*α* = 0.80), Training (*α* = 0.82) and Total scale (*α* = 0.86) had acceptable Cronbach’s alpha coefficients. The subscales were strongly and positively correlated (*r* = 0.62).

**Table 1:** Means, standard deviations, and polychoric correlations of the MCPI items.

	M	SD	Skewness	Kurtosis	1	2	3	4	5	6	7
1. Leadership	5.46	0.94	−2.60	8.77							
2. Reception	5.32	1.34	−2.10	4.60	0.54						
3. People	5.15	1.65	−1.83	3.20	0.65	0.59					
4. Instruction	4.93	2.10	−1.53	1.83	0.56	0.47	0.50				
5. Observation	4.72	3.32	−1.44	0.92	0.58	0.49	0.52	0.63			
6. Feedback	4.57	3.31	−1.18	0.30	0.58	0.55	0.54	0.66	0.79		
7. Facilities	4.87	1.84	−1.26	1.29	0.45	0.47	0.46	0.40	0.37	0.44	
8. Organization	5.16	1.51	−1.78	3.25	0.62	0.61	0.63	0.55	0.58	0.59	0.58

### 3.2 Qualitative analysis

To clarify our statistical demonstration of multicollinearity between ‘observation’ and ‘feedback,’ we examined participants’ free-text answers about strengths and weaknesses of the two variables.

#### 3.2.1 Observation

Sixty-six of 171 answers on observation referred to feedback, either explicitly by using the term feedback, or implicitly by using a synonym or describing behavior that conformed to the term feedback. The texts related the term ‘observation’ to preceptors noting and correcting mistakes students had made while completing tasks, supporting students’ learning from mistakes or (inappropriate) halts during history taking. Some examples included: “if I did not complete something well or correctly, they told me and therefore I was able to learn it”; “I could do the examination more confidently, because I knew that I would be corrected if anything went wrong”; “I continuously received feedback,” “we were assessed while we were doing the tasks; possible mistakes were corrected by our preceptor.” “The doctor observed us and gave feedback.” “She gave advice afterwards but also facilitated the process of taking the medical history.” “I could practice, and we discussed what needed to be improved.”

#### 3.2.2 Feedback

Only 10 answers on feedback did not refer, also, to observation. These 10 exceptions related specifically to the nature of feedback: it was personal or stimulating; the student felt it was warranted; or it helped them learn. The remaining 161 responses explicitly linked the feedback to observation of students’ skills in taking histories. They commented on: the timing of feedback (in front of the patient or afterwards) and whether it was given to a whole group or individuals. Examples included: “after the patient had left, we discussed his most meaningful answers and (the preceptor) repeated several times the useful questions we had asked.” “Our preceptor could point out both positive and negative things in a way which kept us motivated.” “(Feedback) was given not in the presence of the patient but after he had left, therefore we were not humiliated in front of the patient at the beginning.” “We had (feedback) in general during every session, but it was common as well to receive personal feedback.” “We always discussed the patient’s case with the preceptor.” To receive personal feedback on “what I did well during history taking and what I need to improve.”

### 3.3 Evidence of the factorial validity of the MCPI

Bartlett’s statistics and Kaiser-Meyer-Olkin (KMO) tests showed the adequacy of the polychoric correlation matrix to the factor model (*χ*^2^(28) = 2893.6; *p* < 0.001; KMO = 0.906). Only one factor with eigenvalues greater than one emerged (eigenvalue: 4.86, explained variance: 60.9%), and the parallel analysis also confirmed the existence of a unidimensional structure.

**Figure 1:**
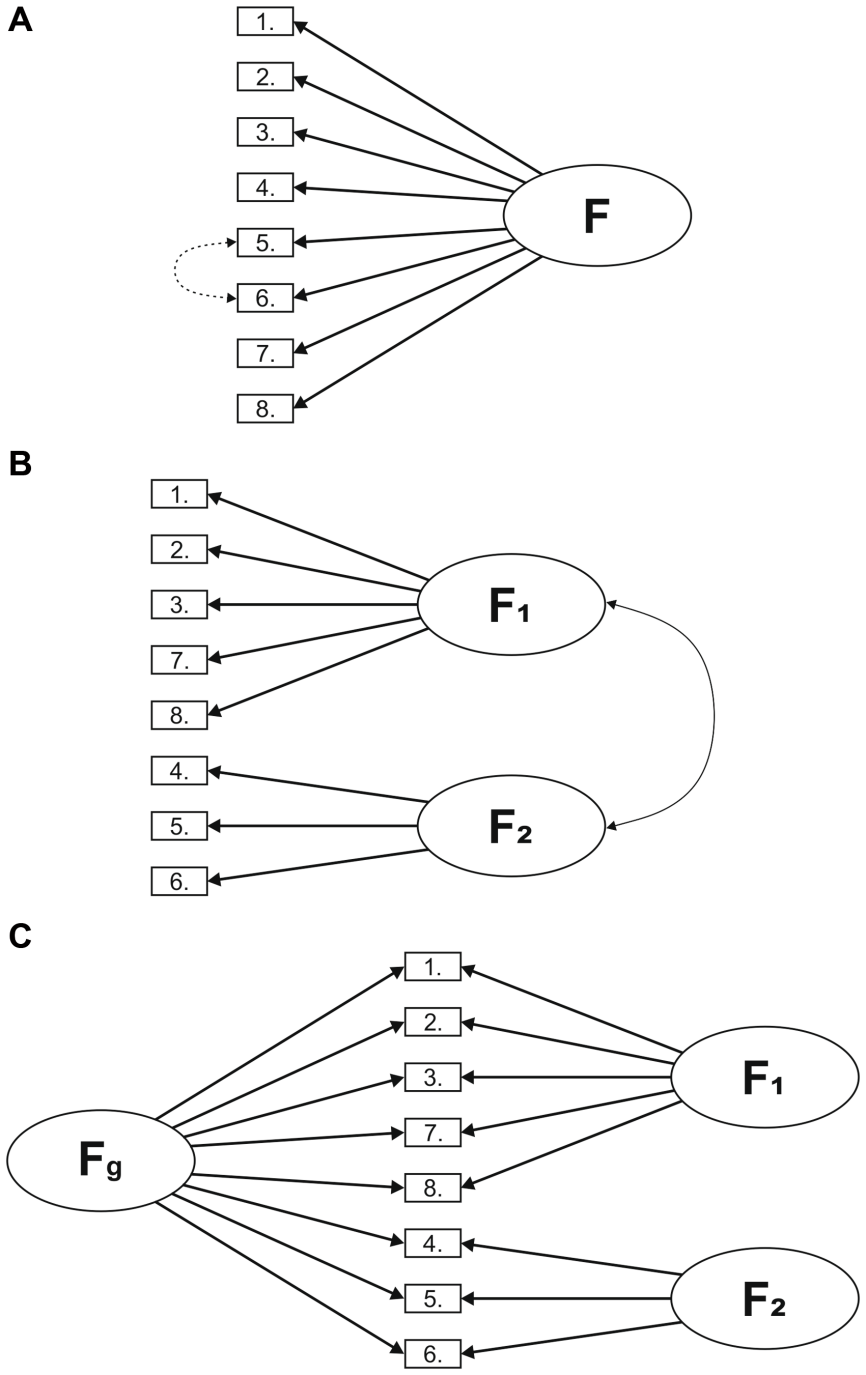
Different factor models of the MCPI. **(A)** Unidimesional **(B)** Two dimensions with correlation **(C)** Bifactor model.

Since the scoring method of the original article of MCPI (10) suggested a bifactor model, where a general factor and two subscales were present, we complemented the EFA by performing a confirmatory factor analysis (CFA) with different models: (A) unidimensional, (B) two dimensions with correlation, and (C) bifactor model (Figure 1).

#### 3.3.1 Overall model fit of the MCPI

First, we presumed a one-factor model based on the results of EFA (Figure 1A). Second, we tested a two-factor model (Learning environment and Training), in which the two factors were distinct but correlated (Figure 1B). Third, based on the previous, we examined a bifactor model, according to which the MCPI comprises a general factor (F_g_) that explains all items, and two factors [Learning environment (F_1_) and Training (F_2_)] that account for the influence of the domains over the general factor (Figure 1C).

As presented in Table 2, the fit of the unidimensional factor structure was adequate though lowest among all possible models. The modification index suggested that adding the error covariances among items 5 and 6 would improve the model fit. Two dimensions with correlation showed acceptable fit, but slightly lower than the modified single factor model. The bifactor model provided an excellent fit to the correlation matrix.

**Table 2:** Goodness of fit statistics for all tested measurement models of the MCPI.

Model	*χ* ^2^	df	CFI	TLI	RMSEA (90% CI)	WRMR
Unidimensional	216.86	20	0.956	0.939	0.122 (0.111, 0.141)	1.220
Modified unidimensional	40.12	17	0.995	0.992	0.047 (0.028, 0.065)	0.486
Two dimensions with correlation	52.80	19	0.992	0.989	0.053 (0.036, 0.071)	0.575
Bifactor model	16.24	12	0.999	0.998	0.024 (0.000, 0.050)	0.284

*χ*^2^, Chi square; df, degree of freedom; CFI, Comparative Fit Index; TLI, Tucker-Lewis Index; RMSEA, Root Mean Square Error of Approximation; WRMR, Weighted Root Mean Square Residual.

#### 3.3.2 Results of the CFA

**Figure 2:**
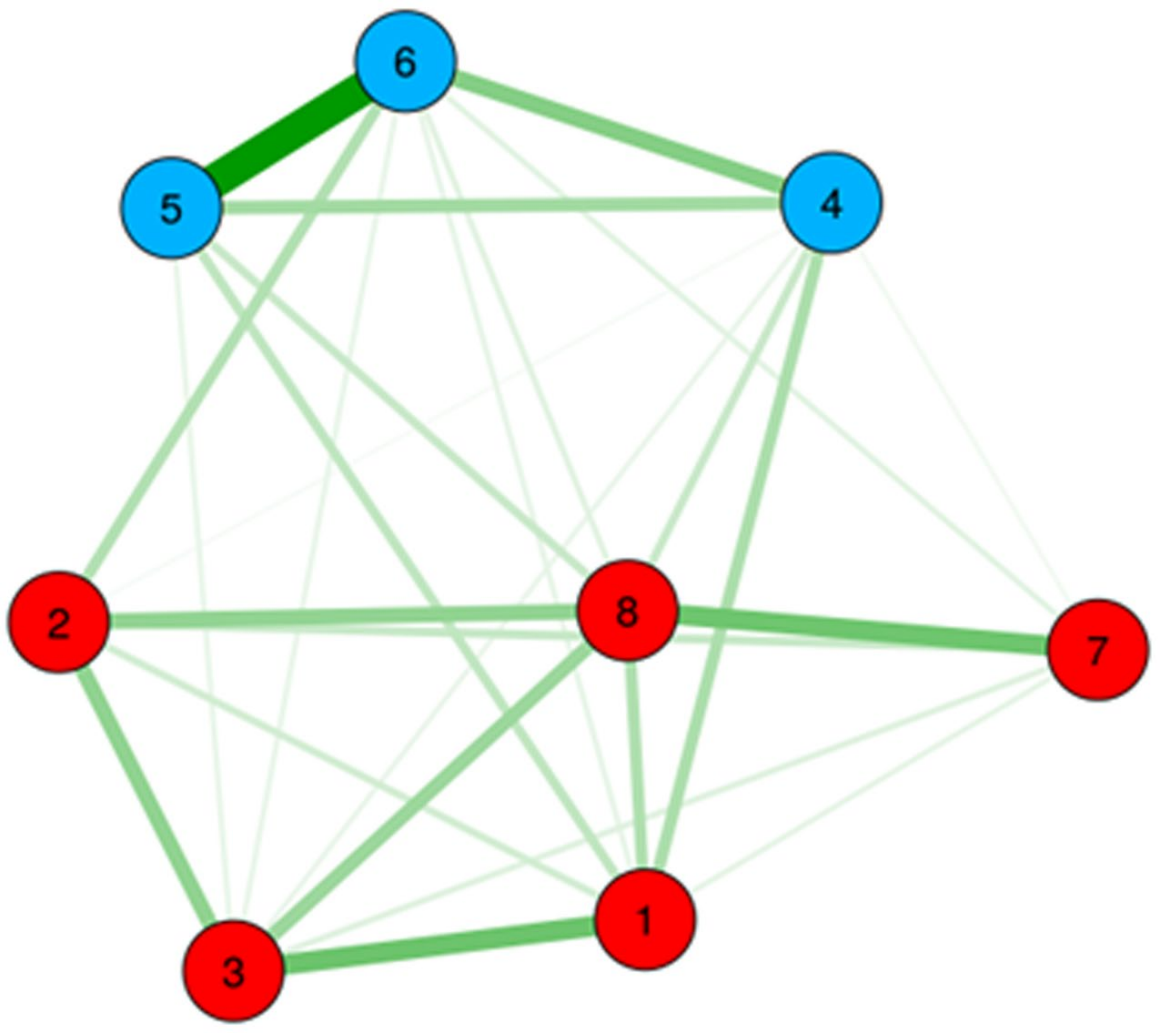
Gaussian graphical model of the MCPI items. Color groupings correspond to higher order dimensions of Learning environment and Training.

As can be seen from Table 3, the pattern of the results suggested a unidimensional model, with all items loading strongly on a single factor (factor loadings ranging from 0.63 to 0.83). Explained common variance (ECV) for the general factor was 0.82, indicating that the general factor explained a high percent of the common variance. In contrast, the ECV was low for the two specific factors (0.04 and 0.14), indicating that these factors explain a lower proportion of the items’ common variance. As seen in Table 3, ω value was 0.93 for the general factor which indicated that the score variance was due to a single factor. The high value of ωH for the general factor (0.87) indicated that the total scores could be considered unidimensional. The low ωH values for the specific factors (0.01 and 0.28) tended to confirm the unidimensional factor structure. Taken together, the high general ECV values and the high value of ωH, indicated unidimensionality, which was consistent with the Exploratory Graph Analysis (EGA) results (Figure 2).

**Table 3:** Confirmatory factor analysis: standardized loadings, explained common variance and model-based reliability estimates for the MCPI.

	Unidimensional	Bifactor CFA			IECV
		*F_g_*	*F_1_*	*F_2_*	
1. Leadership	0.79	0.82	−0.23		0.93
2. Reception	0.74	0.73	0.13		0.97
3. People	0.77	0.77	−0.01		1.00
7. Facilities	0.63	0.60	0.29		0.81
8. Organization	0.83	0.82	0.28		0.89
4. Instruction	0.66	0.66		0.33	0.80
5. Observation	0.68	0.68		0.54	0.61
6. Feedback	0.72	0.72		0.55	0.63
ω		0.93	0.88	0.88	
ωH		0.87	0.01	0.28	
ECV		0.82	0.04	0.14	

CFA, confirmatory factor analysis; ω, omega; ωH, omega hierarchical; ECV, Explained Common Variance; IECV, Individual Explained Common Variance; F_g_, general factor; F_1_, learning environment; F_2_, training.

## 4 Discussion

The main objective of our study was to investigate the internal structure of the Hungarian language version of the MCPI and analyze its reliability. The results of the CFA, as well as the exploratory graph analysis (EGA), supported a bifactor structure. This suggests that the covariation among the items of MCPI may be best explained by a single general learning environment factor (F_g_) that reflects the common variance across all items. Additionally, there were two sub-factors: learning environment, which included items for leadership, reception, people, facilities, and organization; as well as training, which included items for instruction, observation and feedback. This interpretive model captures some unique common variance among clusters of items (31).

### 4.1 Factor structure of the Hungarian version of the Manchester Clinical Placement Index

MCPI was originally developed in an English language undergraduate medical educational context (10), where principal component analysis revealed a bifactor structure in both hospital and community placements (10). The findings were similar when MCPI was translated into Bahasa Indonesian language (32); however, our psychometric analysis of the Hungarian version of MCPI showed that the bifactor solution was compromised by only the general factor giving acceptable model-based reliability; the reliability of the subfactors fell far short of a statistical criterion of plausibility. The EFA alongside the ECV analysis suggested that, from a statistical viewpoint, MCPI may be best conceptualized as a unidimensional measurement tool, the subscale scores being primarily determined by respondents’ overall perception of their learning environment. The finding of a bifactor structure, comprising one interpretable general factor and narrower subfactors, is similar to studies that subjected well-validated clinical instruments to bifactor modeling, like the Beck Depression Inventory-II, the Wechler Adult Intelligence Scale-IV or the Internalized Stigma of Mental Illness Scale (33–35).

### 4.2 Interpreting multicollinearity using mixed-method design

Our observation of multicollinearity between observation and feedback is also worth considering. From a theoretical point of view, both instruction and feedback depend on close observation (36). The qualitative component of this research showed that few students regarded observation and feedback as distinct constructs. Most of their free text answers to the observation item related to preceptors’ feedback on their performance, suggesting that these two behaviors are inseparable from one another, at least in Hungarian students’ experiences. Earlier research in a UK context, however, showed that some clinical educators gave feedback without having directly observed students performing clinical tasks (37). Given this important difference between the present context and the context within which MCPI was originally validated, the multicollinearity might be a context effect. Although use of standard measures (e.g., of health status) is encouraged because it fosters internalization, differences in language, culture and country may confound the meaning of items (38). This could be tested in future research regarding education as well.

### 4.3 The role of textual and numerical information in the implications for medical education assessment and curriculum planning

It is important to consider, from an educational viewpoint, what makes a learning environment measure like MCPI more or less valid. The ability to discriminate reliably between constructs is self-evidently important when high-stakes decisions depend on a measure as, for example, in summative assessments that determine whether a student qualifies as a doctor. MCPI was not designed, however, to make high-stakes assessments. Statistical reliability was only one factor contributing to its validity, for which there is a precedent in clinical as well as educational instruments, which have included items for subjective as well as objective reasons (39). MCPI’s design was informed by education theory (10) as well as empirical research (9, 40). To be specific, Billett’s ‘mutual interdependence’ theory (41) supported the inclusion of self-report items in MCPI. This theory suggests that the quality of learning environments is improved by students’ subjective responses to the affordances of those environments and vice versa in a self-reinforcing feedback loop. Since students’ negative experiences often trigger curriculum reforms (7), and MCPI solicits free text reports as well as numerical ratings of their experiences, its validity as a quality-improvement tool rests on its subjective as well as objective properties. There are firm grounds to use the aggregate measure but we cannot conclude on present evidence whether it is valuable to report back subscale scores. This could be investigated in future research.

### 4.4 Strengths, limitations, and implications for future research

The present study is the first to assess the psychometric properties of MCPI applying a bifactor model, providing more robust and informative results than the one-dimensional or the correlated model. The factorial structure of the Hungarian version of MCPI was best explained by the bifactor model, however; a strong general factor of the learning environment supported the use of the total score instead of the two subscales. This latter finding needs to be further clarified by research using the same bifactorial model. Additionally, our research is the first to provide the opportunity to use a measurement tool to specifically assess the quality of real patient learning in medical education. The ease of its application as well as the options for additional textual answers makes it possible to use this instrument for curriculum development purposes as well, allowing direct feedback on the needs of medical students. Textual answers also make it possible to explore cultural differences between Hungarian and international students, providing valuable insights into factors contributing to the successful internationalization of medical education.

## Data availability statement

The raw data supporting the conclusions of this article will be made available by the authors, without undue reservation.

## Ethics statement

The studies involving humans were approved by Semmelweis University Regional and Institutional Committee of Science and Research Ethics (No. 243/2019). The studies were conducted in accordance with the local legislation and institutional requirements. The participants provided their written informed consent to participate in this study.

## Author contributions

SF: Data curation, Investigation, Project administration, Writing – original draft. SR: Data curation, Writing – original draft, Conceptualization, Formal analysis, Methodology, Software, Validation, Visualization, Writing – review & editing. CH: Data curation, Writing – review & editing, Investigation. PK: Data curation, Writing – review & editing. ÁS: Data curation, Writing – review & editing. KV: Data curation, Writing – review & editing. PT: Writing – review & editing, Resources, Supervision. EN: Supervision, Writing – review & editing, Data curation, Visualization. TD: Supervision, Writing – review & editing, Conceptualization, Writing – original draft. AE: Conceptualization, Supervision, Writing – original draft, Writing – review & editing, Data curation, Investigation, Methodology, Project administration, Software.

## Footnotes

^1^ https://www.medistudents.com/osce-skills/patient-history-taking

^2^ https://appdim.shinyapps.io/app_dimensionality
